# Comprehensive assessment of risk factors for postoperative urinary tract infection following pediatric pyeloplasty

**DOI:** 10.1007/s00383-026-06512-5

**Published:** 2026-06-21

**Authors:** Nikhil V. Batra, Kate H. Adaniya, Ashorne K. Mahenthiran, Zachary Eardley, Mark P. Cain, Martin Kaefer, Kirstan K. Meldrum, Rosalia Misseri, Richard C. Rink, Joshua D. Roth, Benjamin M. Whittam, Konrad M. Szymanski, Pankaj P. Dangle

**Affiliations:** Division of Pediatric Urology, Riley Hospital for Children Indiana University School of Medicine, 535 N Barnhill Dr, Indianapolis, IN 46202 USA

**Keywords:** Ureteropelvic junction obstruction (UPJO), Pyeloplasty, Urinary tract infection (UTI), Complications

## Abstract

**Purpose:**

Variable patterns of perioperative management and postoperative drainage following pyeloplasty are utilized at our institution. We reviewed our patient population and practices to assess risk factors for postoperative urinary tract infection (UTI) following pediatric pyeloplasty.

**Methods:**

Electronic health records of patients who underwent open or robotic pyeloplasty from 2018 to 2023 were retrospectively reviewed. Demographics, perioperative characteristics, and postoperative UTI within 30 days were evaluated. Postoperative UTI was defined as symptomatic and requiring antibiotic therapy. Patients with concurrent conditions elevating risk for UTI and asymptomatic patients with positive cultures were excluded. Univariate and multivariate statistical analysis were performed.

**Results:**

295 patients underwent pyeloplasty with median age of 13 months (IQR 3–91). Eleven (4%) patients had febrile UTI within 30 days of surgery. On univariate analysis, female gender (*p* = 0.007) and UTI history (*p* = 0.045) were significantly associated with risk for postoperative UTI. On multivariate analysis, only female gender (OR 4.5, 95% CI 1.2–17.3, *p* = 0.026) remained significantly associated. No other variables, including type of postoperative drainage and nephrostomy tube drainage prior to surgery, were associated with increased risk.

**Conclusion:**

Female gender and UTI history were significant predictors of postoperative UTI following pediatric pyeloplasty. This knowledge may help inform parents during preoperative counseling.

## Introduction

Open (OP) and robotic-assisted laparoscopic (RALP) pyeloplasty are both commonly used in the surgical management of pediatric patients with ureteropelvic junction obstruction (UPJO). The most common complications of pyeloplasty include urinary tract infections (UTI), wound infection, and urine leak [1]. UTIs occur in 2–15% of patients undergoing RALP and can be affected by a variety of factors such as weight, constipation, dysfunctional voiding, circumcision status in boys, and use of prophylactic antibiotics [2, 3]. Gender and history of UTI are also known to be risk factors for infection. Prior to surgery, many surgeons obtain pre-operative urine cultures to reduce the incidence of UTIs and surgical complications post-operatively, although this practice is not standard and is certainly debated [4, 5]. Techniques for urine collection vary substantially. Sterile bag collections are frequently used due to their less invasive nature but provide a less sterile samples with contamination rates between 30 and 80% [6]. Catheterization and suprapubic bladder aspiration for collection reduces the likelihood of contamination, but these methods are more invasive and can be uncomfortable for patients making them less frequently utilized, especially when obtained locally. Also worthy of note are the potential costs associated with obtaining this additional preoperative testing, which may or may not even be usable.

Variable patterns of preoperative urine culture testing, perioperative management and postoperative drainage following pyeloplasty are utilized at our institution. We aimed to review our patient population and practices and assess risk factors for postoperative UTI following pediatric pyeloplasty to help identify high risk individuals that may benefit more from preoperative urine culture testing.

## Materials and methods

A retrospective review of patients who underwent OP or RALP at our institution between 2018 and 2023 was performed. Electronic health records were reviewed for patient demographics, UTI risk factors, perioperative characteristics, and postoperative UTI within 30 days. Risk factors for UTI investigated were gender, circumcision status, UTI history, and presence of constipation and dysfunctional voiding (DV). UTI was defined by the presence of lower urinary tract symptoms (LUTS) with a positive urinalysis and positive urine culture meeting American Academy of Pediatrics (AAP) diagnostic criteria [7]. Asymptomatic patients with positive cultures were not considered as having UTIs. Patients with a prior history of urinary tract reconstructive surgeries were excluded (i.e. redo surgery).

In regard to surgical pathway, all pyeloplasty cases regardless of approach received perioperative antibiotic prophylaxis, most commonly first-generation cephalosporin, unless an allergy or prior urine culture necessitated the use of an alternative agent. All patients remained admitted overnight for observation with an indwelling foley catheter which was removed on postoperative day 1. Patients typically discharged on postoperative day 1. The use of antibiotic prophylaxis at discharge was a surgeon-dependent practice.

The Fisher’s exact test was used for univariate analysis and multivariate logistic regression was used to evaluate risk factors of significance for postoperative UTI following pyeloplasty as well as effect size. Statistical analysis was performed using State/SE 18.0 (StataCorp. 2023. Stat Statistical Software: Release 18. College Station, TX: StataCorp). Institutional review board approval was obtained for this study (IRB # 1605024102).

As a secondary aim, a simplified cost comparison model was also generated to compare the economic impact of obtaining versus not obtaining preoperative urine cultures prior to undergoing pyeloplasty. Rates of postoperative UTI were derived from the outcomes data in this study with ranges for average antibiotic costs and inpatient admissions for management of UTI with intravenous (IV) antibiotics derived from United States Medicare and Medicaid data [8, 9]. Microsoft Excel was used to create this model (Microsoft Corporation, 2025).

## Results

A total of 295 patients underwent pyeloplasty and were included at a median age of 13 months (IQR 3–91). Surgical approach was open in 176 (59%) and robotic in 121 (41%). Median preoperative hydronephrosis grade was 4 (IQR 3–4) and median clearance half-time of the affected side determined via nuclear renography was 35 min (IQR 19–67). Patients were male in 74% of cases, of which 24% were circumcised. A history of UTI was noted in 7.8% of patients, constipation in 16% of patients and DV in 3% of patients. For postoperative drainage, 130 (44%) of patients had Salle stents utilized versus 168 (57%) with internal ureteral stents. Of those with ureteral stents, 76 (45%) had ureteral stents with extraction strings. Prior to surgery, 36 (12%) of patients had an indwelling nephrostomy tube placed either for infection or to preserve renal function in the setting of massive hydronephrosis. A summary of patient demographics can be seen in Table 1.

**Table 1:** Patient demographics

Patient demographics (*N* = 295)	Value
Age at surgery, median years (IQR)	4 (2–10)
Gender, N (%)	
Male	221 (75%)
Female	74 (25%)
Surgical approach, N (%)	
Open	175 (59%)
Robotic	120 (41%)
Hydronephrosis grade, median grade (IQR)	4 (3–4)
Differential function, median % (IQR)	48 (40–53)
T1/2 (affected kidney), median minutes (IQR)	35 (19–67)
UTI history, N (%)	23 (8%)
Circumcision status, N (%)	53/221 (24%)
Voiding dysfunction, N (%)	8 (3%)
Constipation, N (%)	47 (16%)

Eleven (4%) patients had a febrile UTI within 30 days of surgery. On univariate analysis using Fisher’s exact test, female gender (*p* = 0.007) and UTI history (*p* = 0.045) were significantly associated with risk for postoperative UTI. On multivariate analysis, only female gender (OR 4.5, 95% CI: 1.2–17.3, *p* = 0.026) remained significantly associated. No other variables, including type of postoperative drainage and nephrostomy tube drainage prior to surgery, were associated with risk for postoperative UTI. A summary of the variables analyzed can be seen in Table 2.

**Table 2:** Risk of postoperative UTI

UTI risk factors	Postoperative UTI	No postoperative UTI	*p*-value
Female gender	7/74 (9%)	4/221 (2%)	*0.007
History of UTI	3/23 (13%)	8/272 (3%)	*0.045
Circumcised	1/53 (2%)	3/168 (2%)	> 0.99
Voiding dysfunction	0/8 (0%)	11/287 (4%)	> 0.99
Constipation	3/47 (6%)	8/248 (3%)	0.39
Use of prophylactic antibiotics	10/270 (4%)	1/25 (4%)	> 0.99
Ureteral stent with string	3/76 (4%)	5/92 (5%)	0.73
Indwelling nephrostomy tube	2/36 (6%)	9/259 (3%)	0.63

These two values are bolded and italicized as they are statistically significant values

Univariate analysis based on Fisher’s exact testing

## Cost analysis

We aimed to generate a simplified cost comparison between patients with and without urine culture testing prior to pyeloplasty, accounting for impact on rate of postoperative UTI. In the above cohort, 139 patients had preoperative urine cultures obtained, of which 7 were noted to have a postoperative UTI within 30 days; 157 patients did not have preoperative urine culture testing, of which 4 had postoperative UTI (*p* = 0.36). United States Medicare and Medicaid data were used to determine ranges for the costs for urine culture testing ($10–585), oral antibiotic therapy for bacteriuria ($10–70), and inpatient admission for IV antibiotics for treatment of UTI ($4,700-6,500) [8–10]. Cost ranges were seen to vary with insurance coverages although this may be incompletely captured from this population-based data. We noted a per patient cost of $256.69–982.34 for those who underwent preoperative urine culture versus $119.75-165.61 for those who did not. This would translate to an additional average cost of $136.95–816.73 for patients undergoing preoperative urine culture testing.

## Discussion

In this cross-sectional review of pediatric pyeloplasty patients from our institution, we found female gender and history of prior UTI to be key risk factors for development of postoperative UTI. While female gender is the only variable that remained significant on multivariate analysis, it is worthwhile acknowledging early that statistical powering is challenging in the context of this study that had a low event rate of postoperative UTI, albeit similar to the greater literature. We did not find a relationship between other commonly associated risk factors for UTI in general—bladder and bowel dysfunction or uncircumcised status in boys—although it remains possible these relationships may be observed with greater statistical powering.

While the risk factors we identified for postoperative UTI in this group of patients were not novel findings, they did prompt our group to review our practices on obtaining preoperative cultures. 139 patients (47%) had preoperative urine cultures obtained, of which 43 (30%) were positive and 95% of which were treated. Cultures were obtained from a bag specimen in 20 (47%), nephrostomy tube in 11 (26%), catheterized specimen in 3 (7%), and clean catch in 9 (21%) patients. Considering that most cultures were obtained from bag specimens, it does raise the question of seeing if risk stratification may help direct high-risk patients to more appropriately be targeted for preoperative urine culture testing prior to pyeloplasty. Our 4% rate of postoperative UTI in this cohort is comparable to the rates of UTI found in previous pyeloplasty cohorts of 5% in Sheth et al. [11], and 7.59% in Vidovic et al. [3]. Of note, other studies such as Wang et al. have suggested postoperative UTI rates may reach as high as 37.3% [1].

The cost analysis in this study also supports a reason to think more critically about this practice, given the finding of a higher economic burden to patients that have undergone preoperative testing ($136.95–816.74) versus those that do not in the context of preventing postoperative UTI and the associated costs.

A study by Chan et al., evaluating the utility of perioperative urine cultures and antibiotics yielded similar results, more specifically finding no statistical benefit to postoperative antibiotic use in preventing postoperative UTI [12]. They go on to propose an algorithm to help direct antibiotic use geared towards higher risk patients. While our cohort demonstrated no increased risk of infection in uncircumcised boys or those with prior UTI history on multivariate analysis, our group would still advocate for preoperative urine testing in both of these groups, along with patients with factors considered as higher risk in this study. Modern applications of artificial intelligence have also been employed to help risk stratify UTI risk in pediatric pyeloplasty patients, as well as postulate that UTI in the early postoperative period may predispose to recurrent obstruction [13].

## Limitations of the study

This study has several notable limitations. First, this is a single institution retrospective review. We observed a low event rate of postoperative UTI in this study, which makes obtaining sufficient powering for detecting changes in more than a few variables difficult. In this study, we focused on a select group of variables with strong clinical relevance and established connections to UTI development in pediatric urologic populations but acknowledge that additional factors may contribute to the development of postoperative UTI as well. Ideally, a multi-institutional study would bolster the volume available to detect changes in multiple variables which may be implicated in postoperative UTI following pyeloplasty. While this analysis should be considered exploratory and hypothesis-generating based on norms for statistical power, we believe that our univariate analysis was at least directed towards well-established risk factors for UTI in general and lends support to our findings. Next, this cohort evaluated both OP and RALP as a combined group. While we observed the same risk factors for postoperative UTI despite surgical approach, we acknowledge that these patient populations reflect different age groups with potentially separate risk factors for infection, although we did not detect these changes in our statistical analysis. Finally, the cost analysis used in this study was simple and perhaps fails to capture some elements that may over or understate the costs associated with either approach. Although we considered using more complicated models, we did not feel like those accounting for recurrent or long-term risk (i.e. Markov modeling) were necessary given that we were only looking at short-term postoperative risks. Future studies that are more sufficiently powered are needed to assess the relationship between postoperative UTI following pyeloplasty and long-term need for reoperation.

## Conclusion

Female gender and UTI history were the only significant predictors of postoperative UTI following pediatric pyeloplasty. Knowledge of the cumulative risk in this group may help inform parents on the higher risk stratification for postoperative UTI and preoperative testing.

## Data Availability

Deidentified patient data from our institution was used in this study after institutional review board approval. Thus, the data are not publicly available but may be shared if requested with appropriate institutional oversight and approval.
